# Enhancing In Situ Carbonation of Fresh Paste via Cal-Al Layered Double Oxide and Mixing Parameter Optimization

**DOI:** 10.3390/ma18214943

**Published:** 2025-10-29

**Authors:** Lin Chi, Xulu Wang, Xuhui Liang, Vahiddin Alperen Baki, Jiacheng Zhang, Qiong Liu, Bin Peng, Shuang Lu, Songmao Yang, Min You

**Affiliations:** 1 Xinjiang Key Laboratory of High Value Green Utilization of Low-Rank Coal, Changji 831100, China; chilin@usst.edu.cn (L.C.); lus@hit.edu.cn (S.L.); 2 School of Environment and Architecture, University of Shanghai for Science and Technology, Shanghai 200093, China; 233432038@st.usst.edu.cn (X.W.); lq612@usst.edu.cn (Q.L.); 223382077@st.usst.edu.cn (S.Y.); 2335054826@st.usst.edu.cn (M.Y.); 3 Department of Architecture and Civil Engineering, University of Bath, Bath BA2 7AY, UK; 4 Department of Civil Engineering, Faculty of Engineering, Karadeniz Technical University, Trabzon 61080, Türkiye; valperenbaki@ktu.edu.tr; 5 School of Materials and Chemistry, University of Shanghai for Science and Technology, Shanghai 200093, China; jiachengzhang@usst.edu.cn; 6 School of Civil Engineering, Harbin Institute of Technology, Harbin 150001, China

**Keywords:** cementitious materials, layered double oxide, carbon neutrality, in situ carbonation, sustainable construction

## Abstract

CO_2_ mixing is one of the implementation techniques of carbon capture utilization and storage (CCUS) in concrete to tailor the performance of cementitious materials and reduce the carbon footprint. Therefore, increasing the total amount of carbon capture capacity of cement-based materials has become the key point of recent research. This study investigates the influence of Cal-Al layered double oxide (LDO) and mixing parameters on key properties of cement pastes under CO_2_ mixing, including mechanical performance, microstructure, phase assemblages, and carbon capture capacity. A particular emphasis was placed on evaluating a novel bubble mixing technique, which was developed to enhance the conventional atmospheric mixing process. The results indicate that, compared to the traditional method, bubble mixing reduced the mixing intensity by 10% but increased the effective carbon sequestration capacity by 0.68%. The observed strength reduction after bubble mixing was consistent with higher water adsorption, indicating the formation of a more porous structure. A higher carbon capture efficiency was achieved with bubble mixing compared to atmospheric mixing, as revealed by further investigation. Crucially, the introduction of LDO significantly enhanced the carbon capture capacity, with improvements of up to 34% compared to the groups without LDO. This highlights the substantial potential of LDO in reducing the carbon footprint of cementitious materials and offers a novel insight for enhancing CO_2_ mixing in cement.

## 1. Introduction

Global warming has risen as one of the most pressing challenges worldwide. CO_2_ emissions resulting from human production activities are the major cause and must be mitigated to alleviate the environmental burden [1,2,3]. With the increasing demand for urbanization over the past few decades, the construction industry has emitted 8% CO_2_, largely due to the extensive use of Portland cement as a binder [4]. Consequently, the urgent need to reduce carbon emissions in the construction industry has drawn attention from both academic and industrial sectors.

Nowadays, carbon capture, utilization, and storage (CCUS) technology is a cutting-edge approach aimed at industrial decarbonization [5]. Generally, this technology captures CO_2_ generated during industrial production, compresses it under high pressure, and either stores it underground or utilizes it for product manufacturing to reduce CO_2_ emissions [6,7]. It has been recognized that CCUS technology can be an effective tool to mitigate the carbon footprint of the cement industry. Carbonation curing, for instance, has been reported as one of the major approaches for the implementation of CCUS technology in the construction industry [8]. Cement and concrete can be used as a carbon sink due to ca-bearing hydration products, which favor carbonation by generating calcium carbonate and calcium silicate hydrates (C-S-H) [9]. The reactions can be detailed in the following equations [10]:

(1) $$3CaO\cdot SiO_{2}+(3-x)CO_{2}+nH_{2}O\to xCaO\cdot SiO_{2}\cdot nH_{2}O+(3-x)CaCO_{3}$$

(2) $$2CaO\cdot SiO_{2}+(2-x)CO_{2}+nH_{2}O\to xCaO\cdot SiO_{2}\cdot nH_{2}O+(2-x)CaCO_{3}$$

Concrete subjected to carbonation curing is normally reported with great improvement on early-age strength enhancement and microstructure refinement, as the reaction degrees of the clinker phases can be largely improved under carbonation curing [11]. Despite these benefits, there are shortcomings that need to be addressed. For instance, as carbonation curing requires a relatively long duration, the intensive demands for the carbonation chambers remain challenging for its execution [12]. Meanwhile, for the hardened cement concrete, carbonation curing may lead to a gradient difference in terms of the reaction of cement clinker, which leads to an uneven distribution of carbonated phases [13]. This has the potential to cause undesirable performance variations in cement-based materials.

In addition to carbonation curing, another approach for CCUS in cement-based materials can be the in situ carbonation of cement paste, namely carbonation mixing. Compared to carbonation curing, it has the advantage of reduced time consumption in concrete production, as it is performed during the initial mixing process [14]. Moreover, it eliminates the need for specialized carbonation curing chambers, making it more feasible for industrial applications. Carbonation mixing can also evenly distribute the carbonated phases through the mixing process with the obtaining of more homogeneity of the cement matrix. However, carbonation mixing involves various mixing parameters, which can significantly impact the mechanical properties and carbon capture capacity of concrete. Wang et al. [15] quantified the optimal proportion of CO_2_ injection during the mixing stage (1.6 wt.%) and observed a 36.5% increase in the 3-day compressive strength of the cement paste and a 20% increase in the 28-day strength. These studies have shown that carbonation mixing can possibly enhance the mechanical properties of cement concrete, further underscoring its practical potential. Nonetheless, the carbon capture efficiency of cement pastes by carbonation mixing is reported to be lower than that of long-term carbonation curing [16]. Improving this efficiency is a challenge to industry and academia for reducing the carbon footprint of the cement or concrete industry.

Layered double oxide (LDO) is the calcined product of layered double hydroxides (LDH) and has recently been studied for improving the performance of cementitious materials. In the work by Khalkhal et al. [17], CaAl-LDH was applied as a hardening accelerator in concrete. The specimens containing CaAl-LDH showed a 61% increase in early compressive strength and a 71% increase in flexural strength. Chen et al. [18] studied the influence of LDO on the physical and mechanical properties of cement-based materials and found the mechanical properties of cement mortar with 0.5% and 1% of LDO increased accordingly. Other than its benefits for strength enhancement, it should be highlighted that LDO can reconstruct its structure to form LDH through cement hydration due to its “memory effect”. LDH has been widely reported with desirable capacity for anion capture [19]. Carbonate ions, in particular, are commonly captured within this structure. Chi utilizes the anion exchange capacity of CaAl-LDH and applies it to the immobilization of heavy metals, resulting in a reduction in the leaching rates of Pb^2+^ and Mn^2+^ in cement paste by 7% and 53%, respectively [20]. Duan et al. [21] found that with the addition of Mg_3_Al-CO_3_ hydrotalcite, especially calcined hydrotalcite (LDO), the carbonation depth of concrete was significantly reduced. Meis et al. [22] studied the influence of LDHs’ particle size on the CO_2_ adsorption performance, revealing that decreasing the particle size can lead to a significant increase in CO_2_ adsorption capacity. These findings demonstrate the innovative application of LDH materials, revealing that CaAl-LDO not only readily participates in the formation of cement hydration products compared to MgAl-LDH, but also serves as an additional calcium source to promote calcium carbonate formation, thereby significantly enhancing the CO_2_ storage capacity of cement-based materials.

The application of LDO specifically for in situ carbonation in cement has not yet been systematically investigated, making this a novel and valuable area of study. This study systematically investigates the synergistic effects of key carbonation mixing parameters, including mixing time, CO_2_ injection duration, and mixing methods in combination with LDO on the properties of cement paste. By evaluating their combined impact on mechanical properties, microstructure, and most critically, CO_2_ storage capacity, this study aims to provide a novel and practical strategy for improving carbonation mixing technology in cement-based materials.

## 2. Materials and Methods

### 2.1. Raw Materials

P·I Portland cement according to GB 175-2023 [23] was supplied by China United Cement Corporation (CUCC). The chemical composition determined by X-ray fluorescence (XRF) analysis is presented in Table 1. The Ca/Al ratio of Ca-Al LDO in this study is 2:1, which is determined according to our previous study [24,25]. The physicochemical properties of Ca-Al LDO are detailed in Table 2.

### 2.2. Research Program

**Figure 1:**
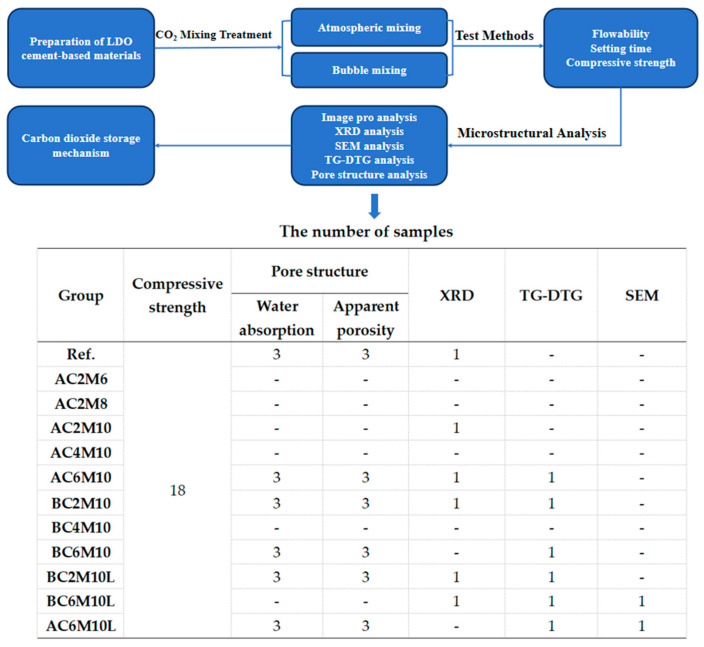
Flow chart of methodology.

The experimental scheme is summarized in Figure 1. The investigation discusses to the effects of CO_2_ mixing parameters and mixing methods on cement pastes, including those containing LDO at the optimal dosage. The detailed procedures for each experimental phase are described in the following section. The table summarizes the number of samples for each group.

### 2.3. Mixture Designs and Sample Preparation

The mixture proportions are listed in Table 3. The water-to-binder ratio was 0.4. To cast pastes, dry powders (cement and LDO) were firstly mixed in the Hobart mixer at a low speed of 60 r/min for 2 min to reach homogeneity. Afterwards, water was added, followed by a high-speed mixing process at 100 r/min. The LDO dosage in this study is 2%, which is determined based on our previous study [25].

Two mixing methods, atmospheric mixing (A) and bubble mixing (B), were studied. Additionally, the impact of varying mixing durations (M) and CO_2_ injection durations (C) was analyzed. Since a rapid loss in flowability was observed during carbonation mixing, the upper limits for CO_2_ injection and mixing times were carefully selected to ensure a smooth casting process. The mixtures are labeled according to these abbreviations, with an ‘L’ added at the end of the name for mixtures containing LDO.

**Figure 2:**
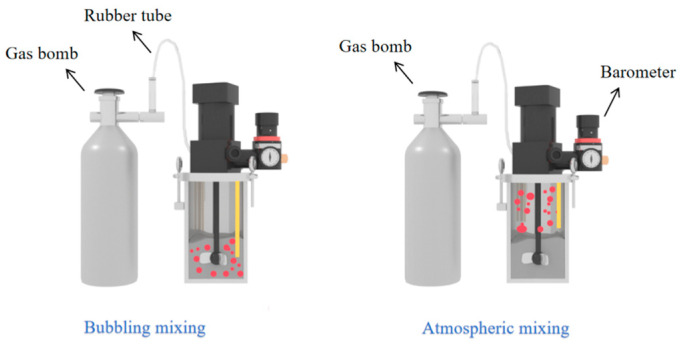
Schematic plot of mixing setup.

For the sample preparation with regard to carbonation mixing, a sealed mixing setup, as presented in Figure 2 was used to ensure that the entire mixing process could be well-controlled through the selected mixing parameters. Two mixing conditions were applied in this research, namely atmospheric mixing and bubbling mixing. In the case of atmospheric mixing, CO_2_ was injected from the top of the mixing container, allowing the gas to initially cover the surface of the cement paste and maintain contact throughout the mixing process. For bubble mixing, rubber tubes were placed at the bottom of the mixer, and CO_2_ was injected directly into the paste during mixing. In this method, the cement paste comes into contact with CO_2_ in the form of gas bubbles. As continuous extension of CO_2_ injection time led to a steady decline in cement paste fluidity (evidenced by gradual thickening and drying), a maximum CO_2_ injection time of 6 min was therefore set to maintain acceptable workability of cement pastes.

### 2.4. Test Methods

#### 2.4.1. Flowability and Setting Time Measurement

The flowability of cement pastes was determined according to ASTM C230/C230M [26]. Cement pastes after mixing were cast into the flow mold until filled, and the mold was lifted vertically and the pastes flowed accordingly. The diameter of the spread paste was measured to determine its flowability. The setting time was measured by Vicat according to ASTM C191-192019 [27]. The test interval between each measurement is within 5 min to ensure an instant measurement.

#### 2.4.2. Compressive Strength

The compressive strength of cement pastes was determined according to ASTM C109/109M-21 [28]. After the mixing process, the paste was cast into cubic molds with dimensions of 2 cm. The samples were covered with plastic film to prevent moisture loss and demolded after 1 day. Subsequently, the samples were cured under standard conditions with a relative humidity above 95% and a temperature of 20 °C until the strength test. Six samples were tested for each sample (loading speed is 1.5 mm/min).

#### 2.4.3. Image Analysis for CO_2_ Bubbles Distribution

CO_2_ gas was injected into the mixer and interacts with the paste in the form of bubbles. The distribution and size of CO_2_ bubbles are closely correlated with mixing parameters and the carbonation process of cement pastes. To investigate this correlation, transparent glue was poured in transparent and sealed mixing containers with a consistent CO_2_ gas flow rate, and a high-speed camera was applied to capture the dynamics of CO_2_ bubbles during mixing. The stirring speeds of 60 rpm for low-speed stirring and 100 rpm for high-speed stirring were applied to observe the impact on bubble dispersion. The bubble size and distribution on the surface of the mixture were recorded and analyzed by image analysis. The stirring process was captured from a consistent angle, with images taken at specific mixing stages: the initial stage (1 min), mid-stage (5 min), and final stage (9 min). Bubble sizes were categorized by pixel area: small bubbles (0–500 pixels), medium bubbles (500–1000 pixels), and large bubbles (1000 pixels or more). Various types of bubble diameter classification are as follows: small bubble 0–2.242 mm, medium bubble 2.242 mm–5.646 mm, large bubble above 5.646 mm. This approach aims to reveal the effect of mixing speed on the CO_2_ bubble size and distribution, which offers insights into the overall carbon sequestration efficiency of cement pastes.

#### 2.4.4. Water Absorption

The water adsorption test was conducted according to ASTM C1585-20 [29]. Cubic samples with dimensions of 4 cm were cured for 3 d, 7 d, and 28 d. The specimens were then vacuum-dried at 60 °C for 48 h until a constant mass was achieved and cooled down to ambient conditions. Subsequently, five sides of each specimen were sealed with epoxy, while the remaining side was exposed to a constant 3 mm water level. The mass change (*M*, in grams) and the corresponding time (*t*, in seconds) were recorded. The capillary water adsorption coefficient (*S*) can be calculated according to Equation (3).

(3) $$\frac{M\left(t\right)}{A}=S\cdot t^{0.5}$$

where A is the surface area of the exposed area (mm^2^).

#### 2.4.5. Apparent Porosity Measurement

The apparent porosity test was conducted according to ASTM C20 [30]. The membranes were first weighed in air, followed by suspending the membranes in water to measure the suspended weight. The membranes were then soaked in water for 24 h, removed from water, and measured again for the soaked weight:

(4) $$\%AP=\frac{w_{S}-w_{A}}{w_{s}-w_{sw}}\times 100\%$$

where *W_A_* is the weight in air(g); *W_S_* is the weight in the water(g); *W_SW_* is the suspended weight (g).

#### 2.4.6. Phase Assemblage

Thin disk pastes were prepared for microstructure analysis. The casting and curing procedures for these samples followed the same protocols as described in Section 2.3. After a predetermined hydration period, the samples were immersed in isopropanol for one week to halt the hydration process. Subsequently, the pastes were vacuum oven-dried at 40 °C for 24 h to remove the isopropanol. The dried samples were then crushed into a powder that passed through a 200-mesh sieve and were used for X-ray diffraction (XRD) and thermogravimetric analysis (TGA).

The XRD test was conducted using a Bruker D8 Advance powder diffractometer (Karlsruhe, Germany). The scanning angles ranged from 5° to 90° of 2 theta, and the scan rate was set to 8° per minute. The carbon capture capacity of the cement pastes was quantified using an STA 650 Thermogravimetric Analyzer (Osaka, Japan). The test was conducted under a nitrogen atmosphere, with temperatures ranging from 30 to 1000 °C with a heat rate at 10 °C/min.

The microstructure of cement pastes was observed by Scanning Electron Microscopy (SEM) using a ZEISS-Sigma 300 (Oberkochen, Germany). The samples were soaked in an ethanol solution for 24 h to terminate hydration and then placed in an oven at 60 °C for 48 h to ensure adequate drying of the fragments. Finally, samples were coated with a thin conductive layer of gold (Au) to minimize charging.

## 3. Results and Discussions

### 3.1. CO_2_ Mixing Parameters on Cement Pastes

Mixing CO_2_ into cement paste can lead to the formation and distribution of carbonate phases initially, which significantly influences the performance of cementitious materials. Technical parameters during mixing, for instance, CO_2_ injection duration, mixing duration, and CO_2_ injection methods, are of great interest. This section investigates these parameters on the performance of cementitious materials, where compressive strength evolution is used as the index for the evaluation, considering its practical engineering significance.

#### 3.1.1. Effects of Mixing Duration

**Figure 3:**
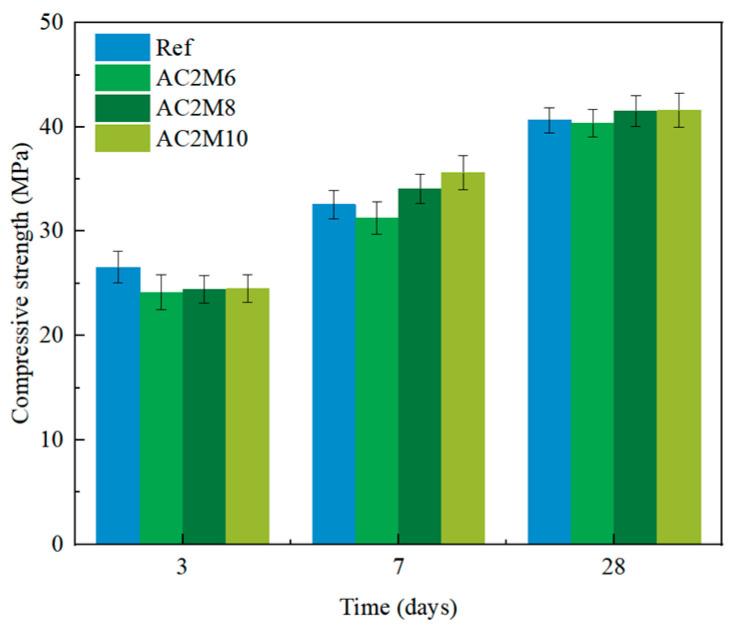
Compressive strength development of cement pastes cast with different mixing times.

Figure 3 illustrates the compressive strength of cement pastes mixed under CO_2_ atmospheric conditions for various durations. At 3 days, a slight reduction in compressive strength is observed in the samples subjected to CO_2_ atmospheric mixing compared to the reference samples. This trend aligns with findings from Liu et al. [31], who reported a reduction in early strength for cement pastes undergoing CO_2_ mixing. However, as the curing period increases, the negative impact of CO_2_ mixing on compressive strength diminishes. In addition, while maintaining the carbonation injection duration for 2 min but extending the mixing duration to 8 and 10 min, a slight improvement in compressive strength at a later age is found. This increase is likely due to a more uniform distribution of the carbonate phases, which contributes more effectively to strength development. Therefore, extending the mixing duration is recommended, provided the paste consistency can still be preserved.

#### 3.1.2. Effects of CO_2_ Injection Duration

**Figure 4:**
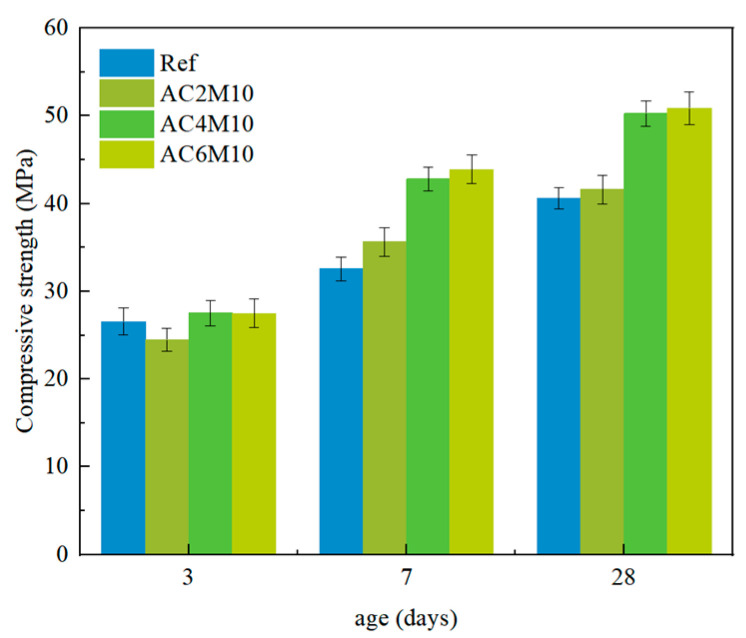
Compressive strength development of cement pastes cast with different CO_2_ contacting durations.

As discussed in Section 3.1.1, extending mixing duration can be beneficial for strength development of cement paste at a later stage; the mixing duration was kept at 10 min thereafter. The impact of CO_2_ injection duration on compressive strength of cement pastes can be found in Figure 4.

In comparison to mixing duration, CO_2_ injection duration has a more pronounced influence on strength enhancement. At 3 days, only the AC2M10 sample exhibits a slight reduction in compressive strength compared to the reference. Extending the CO_2_ injection duration does not negatively impact the early strength but significantly improves the later strength. Notably, increasing the CO_2_ injection time to 6 min results in a 25.2% increase in compressive strength at 28 days.

The CO_2_ injection duration directly correlates with the amount of CO_2_ supplied during the mixing process. Prolonged injection allows more CO_2_ to react with the cement paste, leading to the formation of a greater quantity of carbonate phases. These phases can act as nucleation sites for further hydrate growth, which explains the observed positive effect on strength development [13,14]. However, excessive extension of the CO_2_ injection duration is undesirable, as it also causes a rapid loss of workability in the paste, as observed in current research as well as reported elsewhere [17,32], potentially leading to complications in the subsequent mixing and casting processes.

#### 3.1.3. Effects of CO_2_ Mixing Methods

**Figure 5:**
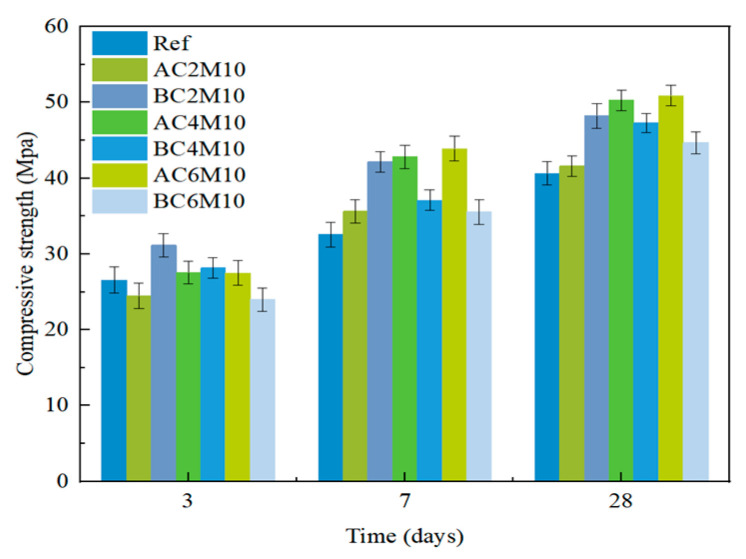
Compressive strength of cement pastes cast with different CO_2_ injection methods.

CO_2_ injection approaches can be a factor influencing the performance of cementitious materials. Figure 5 compares the strength evolution of cement pastes cast under different CO_2_ mixing methods, with a constant mixing duration of 10 min to ensure paste homogeneity. It is interesting to note that when the CO_2_ injection duration is relatively short, bubble mixing results in greater strength in cement pastes compared to atmospheric mixing. For instance, BC2M10 is found to have a higher compressive strength than AC2M10, with an increment of 27.2% at 3 days and 16.0% at 28 days. However, in contrast to atmospheric mixing, where a gradual increase in cement strength occurs with prolonged CO_2_ injection, pastes produced from bubble mixing exhibit a steady decline in compressive strength with extended CO_2_ injection duration.

**Figure 6:**
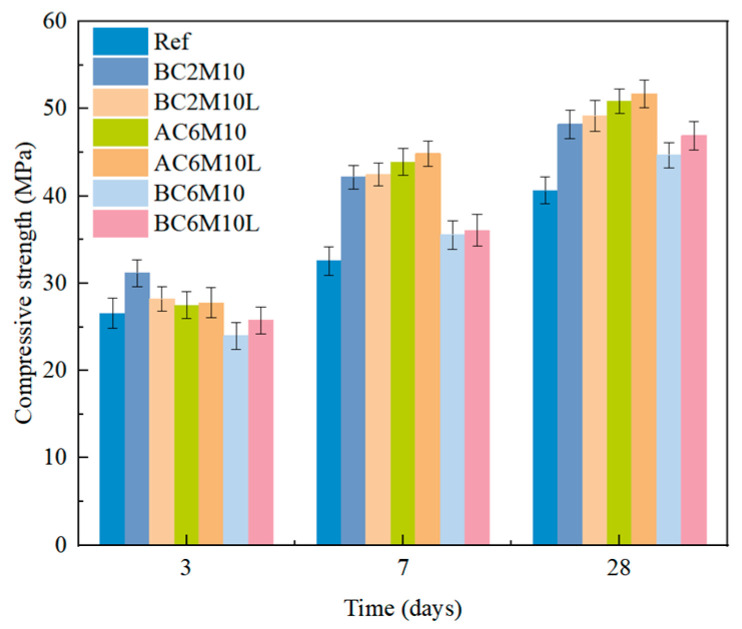
Comparison of strength development of cement pastes containing LDO cast with different CO_2_ injection methods.

For cement pastes containing LDO with a dosage at 2%, as shown in Figure 6, pastes generally show a slight strength improvement under CO_2_ mixing compared to the pure cement pastes, regardless of the mixing methods used. This brings a positive aspect for engineering practice in introducing LDO in cement for carbonation mixing. Similarly, for pastes with 2% LDO, unlike CO_2_ atmospheric mixing, extending the CO_2_ injection duration in bubble mixing tends to have a negative impact on strength development.

As discussed in Section 3.1.2, a longer CO_2_ injection duration leads to increased carbonate formation due to the greater availability of CO_2_ reacting with the cement paste. Despite this, bubble mixing proves to be more effective in facilitating CO_2_ reactions with the cement paste, as evidenced by the higher strength observed in the BC2M10 sample compared to the AC4M10 sample, while being comparable to the AC6M10 sample. The observed decrease in compressive strength with extended CO_2_ injection durations in bubble mixing can be attributed to the excessive presence of gas bubbles within the matrix, which may disrupt the structural integrity of pastes with voids that affect their strength development. During the process of bubble mixing, a layer of solid carbon reaction products is formed on the surface of the particles. This layer functions to segregate the unreacted cement, thereby impeding the hydration reaction [16,33]. During atmospheric mixing, the main carbon sequestration reaction occurs only in the portion of the contact surface due to the weak carbon trapping capacity; therefore, the extension of the aeration time has little effect on the final strength [34,35]. Temperature and humidity have dual effects on cement-based carbon sequestration. Optimal temperature or humidity enhances reaction kinetics and carbonation efficiency, while excessive or insufficient temperature or humidity impairs process viability through moisture loss, workability issues, or diffusion barriers, necessitating parameter optimization [36,37]. Further study would be conducted to explore the effects of temperature and humidity.

**Figure 7:**
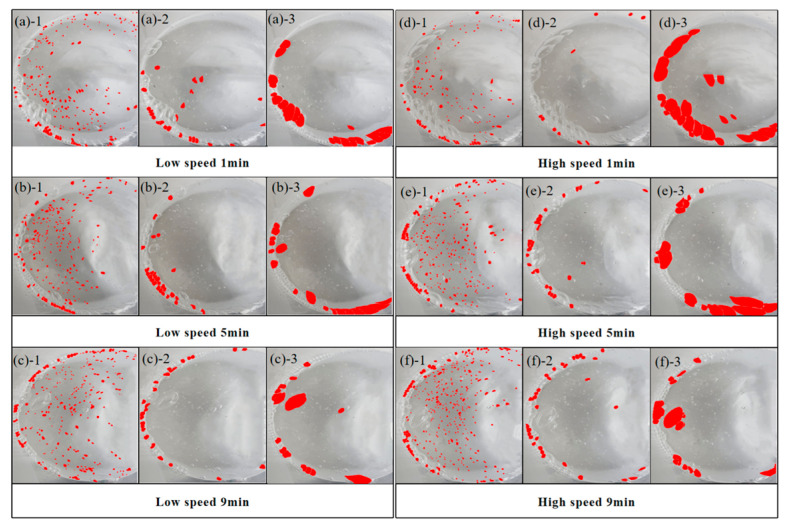
Visualization of gas bubble distribution in a mimicked cement paste during mixing with different mixing speeds (**a**/**b**/**c**)-1 the distribution of small gas bubbles at 1/5/9 min; (**a**/**b**/**c**)-2 the distribution of medium gas bubbles at 1/5/9 min; (**a**/**b**/**c**)-3 the distribution of large gas bubbles at 1/5/9 min; (**d**/**e**/**f**)-1 the distribution of small gas bubbles at 1/5/9 min; (**d**/**e**/**f**)-2 the distribution of medium gas bubbles at 1/5/9 min; (**d**/**e**/**f**)-3 the distribution of large gas bubbles at 1/5/9 min.

**Figure 8:**
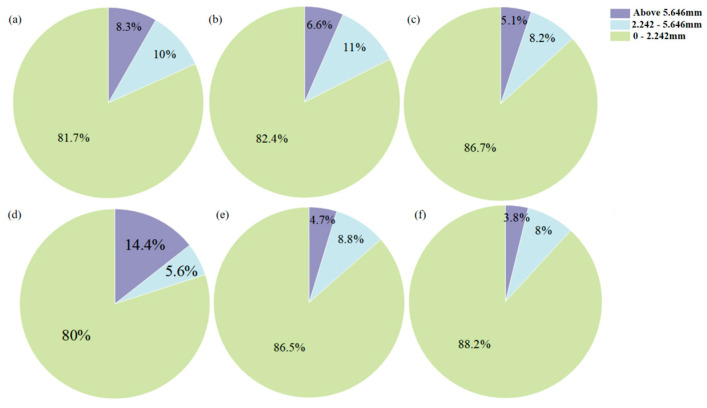
Calculated gas bubble size distribution in the mimicked cement pastes (**a**) low speed 1 min; (**b**) low speed 5 min; (**c**) low speed 9 min; (**d**) high speed 1 min; (**e**) high speed 5 min; (**f**) high speed 9 min.

To illustrate the distribution of CO_2_ bubbles during bubble mixing and the correlation between bubble distribution and mixing speed, an experimental setup using mimicked cement paste was employed. This setup involved recording the bubble dynamics with a high-speed camera. The results are presented with image analysis in Figure 7 and data interpretation in Figure 8. In general, the volume of small gas bubbles is greater than that of medium and large bubbles in all samples. This is due to CO_2_ gas injected through rubber tubes tending to break down into smaller bubbles during the mixing process. By extending the mixing duration, the volume of small bubbles would increase while the other two types of bubbles decrease [38]. This phenomenon occurs at both low and high mixing speeds. However, higher mixing speeds are more effective at breaking larger bubbles, resulting in a better distribution of smaller bubbles, as illustrated in Figure 8 [39]. This improved distribution can be advantageous for the carbonation reaction, as it increases the surface area of the cement pastes in contact with CO_2_ particles [40]. Therefore, for bubble carbonation mixing of cement paste, a high-speed mixing process is highly recommended.

### 3.2. Capillary Water Absorption

**Figure 9:**
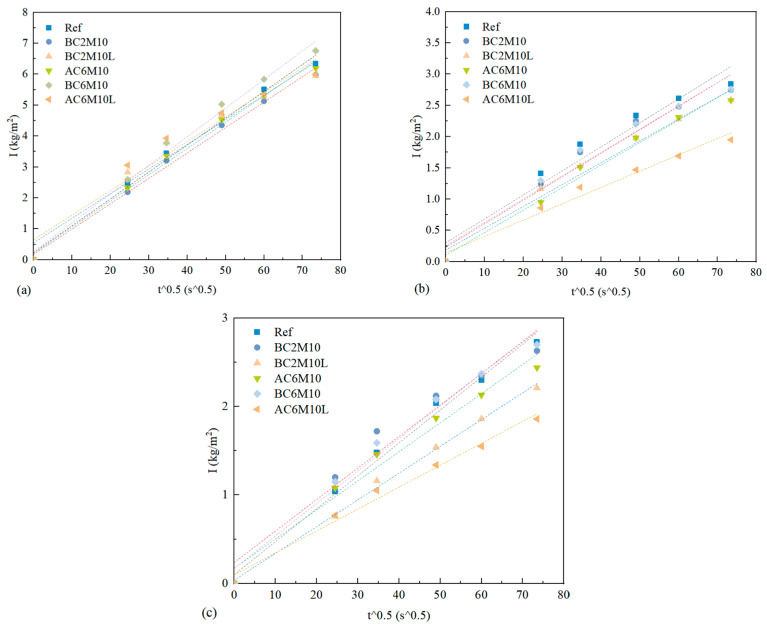
The capillary water absorption of cement pastes (**a**) 3 days; (**b**) 7 days; (**c**) 28 days.

Figure 9 shows the capillary water absorption of cement pastes at 3 days, 7 days, and 28 days. Due to the surface tension of capillary pores, a gradually increasing amount of water was adsorbed in the cement pastes with time. By conducting the liner fitting, the capillary water absorption coefficient of each mixture can be obtained, with detailed parameters shown in Table 4. A greater coefficient indicates a faster water adsorption rate of cement pastes, which should correspond to a higher connectivity of pore structure and possibly a higher porosity [41].

For samples cured for 3 days, the capillary water absorption coefficient ranges from 0.0794 (AC6M10L) to 0.09264 (BC6M10). It is evident that bubble mixing leads to a higher capillary water absorption coefficient compared to atmospheric mixing. Additionally, the introduction of LDO reduces the capillary water absorption coefficient, indicating that LDO may contribute to refining the microstructure of cement pastes [42,43].

As curing progresses, the capillary water absorption coefficient decreases significantly across all samples, irrespective of the CO_2_ injection method. This reduction is attributed to the ongoing refinement of the microstructure as hydration advances [44]. By 7 and 28 days, pastes prepared with carbonation mixing consistently display a lower capillary water absorption coefficient compared to the reference samples. This indicates that CO_2_ mixing enhances later hydration stages, as the formation of carbonate whiskers accelerates the reaction, further refining the pore structure of the cement pastes.

### 3.3. Apparent Porosity

**Figure 10:**
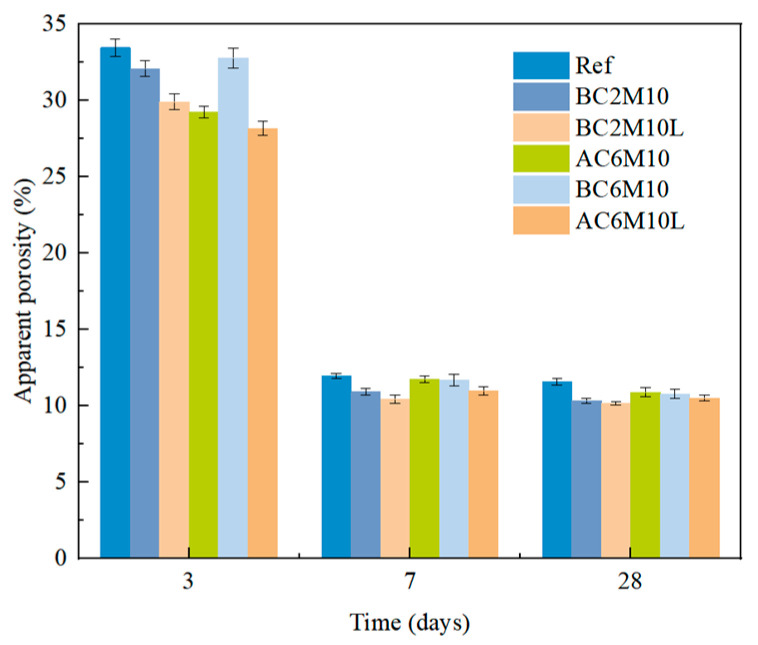
Apparent porosity of cement pastes at 3 days, 7 days, and 28 days.

The apparent porosity of cement pastes is shown in Figure 10. Pastes with CO_2_ mixing are found with a lower apparent porosity, whereas atmospheric mixing is a more significant apparent porosity reduction. The significant apparent porosity reduction from 3 days to 28 days is attributed to the continuous hydration of cement pastes. LDO can further slightly reduce the apparent porosity of cement pastes mixed with CO_2_ [43]. These findings are, in general, consistent with the strength development. Bubble mixing processes may generate numerous fine pores within the paste matrix. Although the transport rates of these pores are significantly lower than those of macropores, they still absorb a small amount of water, thereby influencing the capillary water absorption coefficient [45].

### 3.4. Microstructure Analysis

#### 3.4.1. XRD Analysis

**Figure 11:**
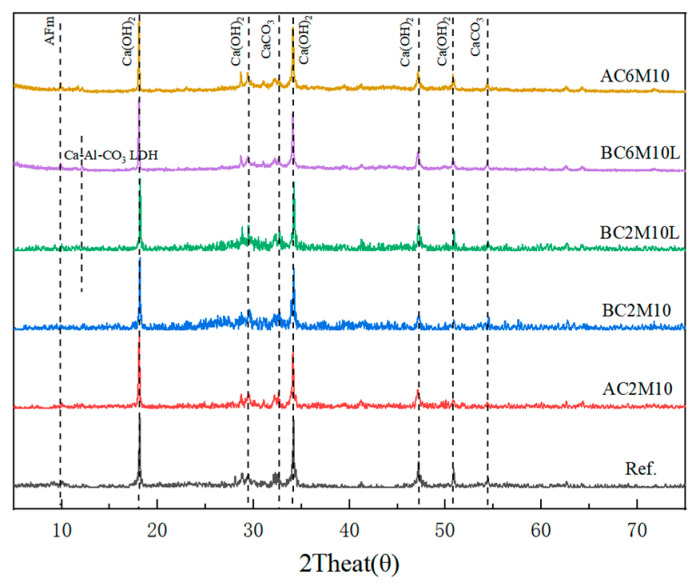
XRD patterns of cement pastes mixed under in situ carbonation with/without LDO.

Figure 11 depicts the XRD patterns of cement pastes under CO_2_ mixing, with the minerals assigned accordingly. In general, the diffraction patterns of all CO_2_-mixed pastes are similar, regardless of the CO_2_ injection methods and the introduction of LDO. The primary phases that can be identified are monosulfate, monocarbonate, portlandite, anhydrous clinker phases, and calcite. Notably, calcite is distinctly recognized as the only calcium carbonate phase, whereas its polymorphs aragonite and vaterite are not observed. The diffraction peak of monocarbonate in plain cement by CO_2_ mixing is very weak, which is due to the slight carbonation of monosulfate. For LDO-containing pastes, on the other hand, the diffraction peak of monocarbonate is more pronounced. However, in LDO blended cement pastes prepared from CO_2_ mixing, the diffraction peak of monosulfate becomes more visible. BC6M10L, for instance, can be clearly seen with the monosulfate peak. This occurs when carbonate ions are incorporated into the LDH structure to form monocarbonate, allowing the sulfate previously adsorbed by Ca-Al LDH to reconstitute monosulfate. At 2θ = 11.6°, the characteristic LDH peaks existed in the BC6M10L and BC2M10L samples, which indicated that CaAl-LDO transformed to Ca-Al-CO_3_ LDH by the “memory effect” in the carbon sequestration reaction [46].

#### 3.4.2. SEM Analysis

**Figure 12:**
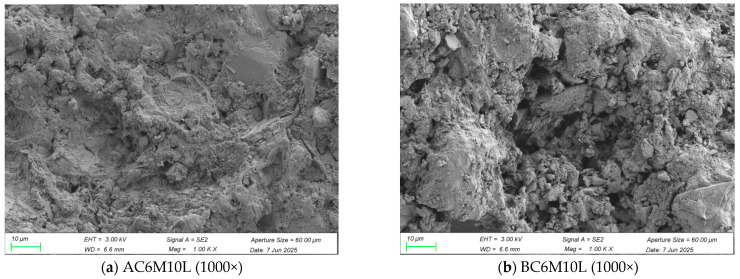

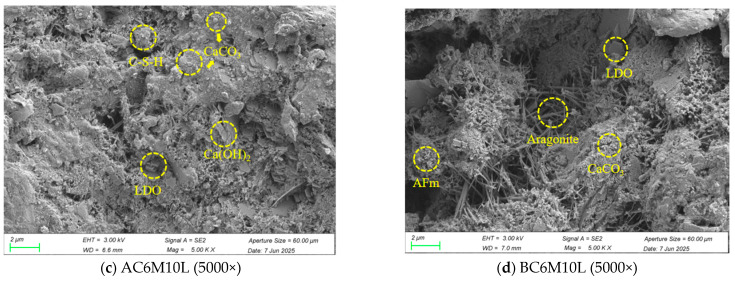
SEM images of cement pastes at 3 days.

Figure 12 presents SEM images of cement pastes prepared using atmospheric mixing and bubble mixing methods, both incorporating LDO. Figure 12a,b compare the morphologies of samples prepared by atmospheric and bubble mixing, respectively, at 1000× magnification under identical conditions. It can be observed that the paste produced by bubble mixing exhibits significantly higher porosity, whereas the atmospheric mixing sample displays a denser microstructure. This contrast becomes more evident at higher magnifications. Figure 12c shows the AC6M10L sample at 5000× magnification [25]. Characteristic hydration products such as C-S-H and portlandite are identifiable by their typical fibrous texture and hexagonal plate morphology, respectively. Additionally, LDH phases can be observed as crystalline structures with rounded plate-like edges. In the BC6M10L sample, at 5000× magnification in Figure 12d, a clearly visible pore structure with a honeycomb-like (faveolate) distribution is evident, suggesting that bubble mixing promotes the formation of fine capillary pores. This microstructural feature helps explain the lower compressive strength observed in the bubble-mixed samples compared to those mixed atmospherically.

#### 3.4.3. TGA

**Figure 13:**
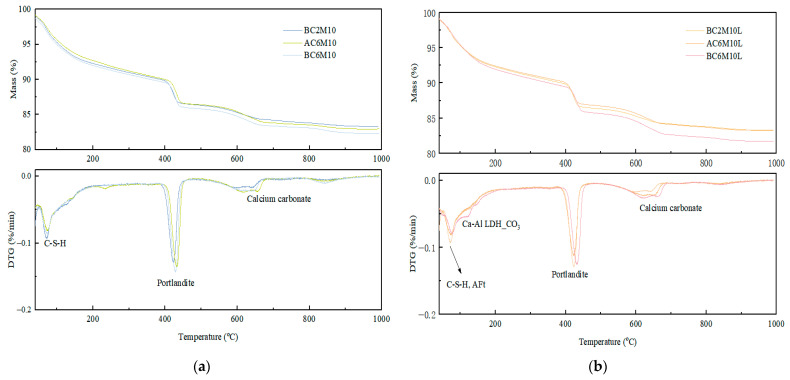
TG-DTG curves of cement pastes (**a**) CO_2_ mixed paste; (**b**) CO_2_ mixed LDO-blended paste.

To evaluate the carbon capture capacity of carbonation-mixed cement pastes containing LDO, TG-DTG analysis was performed, with the results presented in Figure 13. Based on the mineral compositions obtained from XRD, the DTG peaks can be assigned to the corresponding hydrates and carbonate-bearing phases accordingly. Specifically, the decomposition within 120 °C should be largely attributed to the decomposition of C-S-H gels. It is clear that for samples containing LDO, a more pronounced shoulder appears at around 160 °C, which should correspond to the decomposition of LDH-carbonate phases [47], which aligns with the XRD analysis. This highlights the role of LDO in enhancing carbon capture capacity. The peaks observed between about 400 °C and 470 °C are linked to the dehydroxylation of portlandite, while the subsequent decomposition is associated with calcium carbonate phases [31,48,49].

The carbon capture capacity of cement pastes subjected to CO_2_ mixing was calculated based on the mass loss observed during different decomposition stages. Since P.I cement contains only clinker phases and gypsum, the carbonate content of the reference mixture is considered negligible [50,51,52,53]. However, in mixtures exposed to CO_2_ mixing, distinct carbonate phases were identified and attributed to specific decomposition stages. In this research, we follow the decomposition stages proposed by Morandeau et al. [51], who classified five decomposition stages and their associated chemical signals. Using this method, the total CO_2_ binding for each mixture was calculated, as shown in Table 5.

It is observed that both bubble mixing and atmospheric mixing effectively enhance carbon capture, with bubble mixing showing a slight advantage. Furthermore, the addition of LDO further improves the carbon capture capacity, and notably, the combination of LDO and bubble mixing exhibits the highest carbon capture efficiency. By comparing BC6M10 and BC6M10L, it is seen that the introduction of LDO improves the carbon capture by 34.01%. The enhanced carbon capture efficiency can be ascribed to the uniform distribution of CO_2_ gas bubbles in cement pastes, facilitating rapid carbonation, and the robust carbonate adsorption ability of LDO, which forms Ca-Al-CO_3_ LDH with excellent carbonate adsorption capabilities.

### 3.5. Brief Summary

Characteristic parameters of this study are summarized in Table 6, presenting compressive strength, apparent porosity, and CO_2_ storage capacity. At 28 days, the compressive strength of cement-based materials gradually increased with extended CO_2_ injection time under atmospheric mixing, while a decreasing trend was observed under bubble mixing. Among the groups tested at 28 days, AC6M10 under atmospheric mixing exhibited the highest compressive strength, showing an increase of 25.1% compared to the control group. In contrast, under bubble mixing, BC2M10 achieved the highest strength, which was 18.6% higher than that of the control. The incorporation of LDO enhanced the carbon storage performance of the cement-based materials, enabling a maximum CO_2_ storage of 3.31% by cement mass at 3 days.

## 4. Conclusions

CO_2_ mixing is one of the effective methods for reducing the carbon footprint of cement concrete. Minerals with great carbonate binding capacity are of great interest to promote the carbon capture of cementitious materials. Owing to the memory effect of LDO, it is already acknowledged for desirable anion capture and adsorption capacity in various literatures. In this paper, we investigated CO_2_ mixing in cement pastes, where LDO was introduced to promote the carbon capture capacity. Different mixing parameters were systematically studied to provide a comprehensive understanding of the relationship among the performance of cementitious materials, CO_2_ mixing parameters, and LDO. Based on the results, the following conclusions can be drawn:

(1) In atmospheric CO_2_ mixing, extending the CO_2_ injection duration significantly improves the compressive strength. In contrast, extending CO_2_ injection duration leads to a decrease in strength in bubble mixing. While the reaction mechanism remains similar, as indicated from XRD results, the loss in strength in bubble mixing is attributed to the capture of gas bubbles in the matrix, resulting in a more porous microstructure compared to the ones by atmospheric mixing, as evidenced by the higher water adsorption coefficient in bubble-mixed samples.
(2) Bubble mixing is more efficient in carbon capture for cementitious materials, compared to atmospheric mixing, as a consequence of increased contacting surface area between CO_2_ and pastes. This can be validated by the significant early strength improvement by comparing BC2M10 with AC2M10 and can also be supported by the TGA.
(3) The introduction of LDO is evidenced with a promoted carbon capture capacity of cement pastes through carbonation mixing, regardless of the mixing methods. Under the same mixing parameters, the carbon capture capacity can be improved at most by 34.01% with the introduction of LDO, highlighting its significant environmental benefits.

In our current research, LDO shows its outstanding performance in improving the carbon storage capacity of cementitious materials. LDO with different Ca/Al ratios on the performance of cementitious materials, including their dispersion, macroscale properties, and microstructure, as well as carbon capture capacity, are of great interest, meriting significant attention for future research. It is important to note that this study utilized laboratory-synthesized LDO with a controlled particle size. Consequently, potential challenges related to its large-scale, cost-effective production and uniform dispersion during industrial-scale concrete mixing were not investigated. Furthermore, the long-term durability and stability of LDO within the cement matrix under various environmental conditions remain to be verified. Atmospheric mixing and bubble mixing technology can be applied in the field of prefabricated buildings. During mixing, engineered nozzles can uniformly inject carbon dioxide into the concrete, ensuring thorough mixing with the raw materials. Furthermore, concrete mixer trucks can be equipped with an auxiliary CO_2_ cylinder. A specialized gas distribution system integrated into the mixing blades would disperse CO_2_ uniformly throughout the concrete during transit. This facilitates homogeneous carbonation reactions throughout the mixture, effectively implementing both techniques.

## Figures and Tables

**Table 1:** Chemical composition of cement (wt.%).

SiO_2_	Al_2_O_3_	CaO	Fe_2_O_3_	SO_3_	Na_2_O	MgO	f-CaO	LOI (1000 °C)
20.82	4.40	63.34	3.27	2.42	0.59	2.88	0.88	1.54

**Table 2:** General physicochemical properties of Ca-Al LDO.

Property	Parameter
CaO content (wt.%)	40–43.5
Al_2_O_3_ content (wt.%)	17–19
CaO/Al_2_O_3_ (Molar ratio)	4.0–4.5
d_50_ (µm)	5
Heavy metal concentration (ppm)	10
pH value (20 g/L suspension)	7–9
Moisture loss (%) at 105 °C	1
Specific surface area (m^2^/g)	10

**Table 3:** Mixture designs of CO_2_ mixing of cement pastes.

Group	Cement (g)	Water (g)	LDO (g)	CO_2_ Injection Time (min)	Mixing Time (min)	CO_2_ Mixing Methods
Ref.	500	200	-	-	6	-
AC2M6			-	2	6	Atmospheric mixing
AC2M8				2	8	
AC2M10				2	10	
AC4M10				4		
AC6M10				6		
BC2M10				2		Bubble mixing
BC4M10				4		
BC6M10				6		
BC2M10L	490		10	2		
BC6M10L				6		
AC6M10L				6		Atmospheric mixing

**Table 4:** Capillary water absorption coefficient (kg·m^−2^·s^−0.5^) obtained by linear fitting.

	Ref	BC2M10	BC2M10L	AC6M10	BC6M10	AC6M10L
3 days	0.08688	0.08217	0.0796	0.08483	0.09264	0.0794
7 days	0.03844	0.0376	0.03493	0.03603	0.03729	0.02613
28 days	0.0372	0.03562	0.03026	0.03289	0.03674	0.02478

**Table 5:** Results of TGA and potential CO_2_ capture capacity of cement pastes.

	Mass Loss (%)					Chemical Species (%)				
	110–270 °C	270–420 °C	420–480 °C	480–600 °C	600–1000 °C	H_2_O _cem_ + LDH	H_2_O _cem_	CO_2 LDH_	CO_2 cem_	Total CO_2_
BC2M10	3.28	3.08	1.52		2.05		6.36		2.05	2.05
BC2M10L	3.45	3	1.95	1.12	1.98	7.57	6.23	0.23	1.98	2.21
AC6M10	3.45	2.52	2.66		2.46		5.97		2.46	2.46
AC6M10L	3.36	2.93	1.74	1.16	2.42	7.45	5.85	0.27	2.42	2.63
BC6M10	3.59	2.28	2.86		2.47		5.87		2.47	2.47
BC6M10L	3.9	2.35	2.88	1.18	2.84	7.43	5.75	0.29	2.84	3.31

**Table 6:** Results of compressive strength, apparent porosity, and carbon capture capacity of cement pastes.

Group	Compressive Strength (MPa)			Apparent Porosity (3 d)	CO_2_ Storage (3 d)
	3 d	7 d	28 d		
Ref.	26.57	32.57	40.65	33.46%	-
AC2M10	24.49	35.63	41.6	-	-
AC4M10	27.55	42.82	50.29	-	-
AC6M10	27.51	43.91	50.88	29.26%	2.46%
BC2M10	31.16	42.18	48.24	32.07%	2.05%
BC4M10	28.18	37.11	47.29	-	-
BC6M10	23.98	35.57	44.7	32.78%	2.47%
BC2M10L	28.21	42.45	49.15	29.91%	2.21%
BC6M10L	25.76	36.1	46.94	-	3.31%
AC6M10L	27.8	44.86	51.73	28.17%	2.63%

## Data Availability

The original contributions presented in this study are included in the article. Further inquiries can be directed to the corresponding authors.

## Abbreviations

The following abbreviations are used in this manuscript:

CCUS	Techniques of carbon capture, utilization, and storage
C-S-H	Calcium silicate hydrates
LDO	Cal-Al layered double oxide
LDH	Layered double hydroxides
XRF	X-ray fluorescence
